# Post-Synthetic Modification of an Amino-Functionalized Metal–Organic Framework for Highly In Situ Luminescent Detection of Mercury (II)

**DOI:** 10.3390/nano13202784

**Published:** 2023-10-17

**Authors:** Chen Ji, Li Pei, Junyi Qin, Pengyan Wu, Nuo Su, Ting Zhang, Yexin Zhang, Jian Wang

**Affiliations:** Jiangsu Key Laboratory of Green Synthetic Chemistry for Functional Materials, School of Chemistry and Materials Science, Jiangsu Normal University, Xuzhou 221116, China

**Keywords:** metal–organic frameworks (MOFs), fluorescent sensor, post-synthetic modification (PSM), sulfur-containing, mercury (II)

## Abstract

A sulfur-containing metal–organic framework, donated as UiO-66-NSMe, was prepared by the post-synthetic modification (PSM) of UiO-66-NH_2_ with 2-(Methylthio)benzaldehyde, and the successful synthesis of PSM was confirmed by X-ray photoelectron spectroscopy (XPS), FT-IR and ^1^H NMR studies. According to the characteristics of mercury thiophilic, UiO-66-NSMe could be used as a luminescent sensor for Hg^2+^ detection with a high selectivity and sensitivity (*K*_sv_ = 2.5 × 10^4^ M^−1^; LOD = 20 nM), which could be attributed to the coordination between sulfur sites and Hg^2+^ based on XPS results. In practical applications, UiO-66-NSMe yielded satisfactory recovery rates (ranging from 96.1% to 99.5%) when it was employed for detecting Hg^2+^ in spiked environmental samples. Furthermore, UiO-66-NSMe was successfully employed to detect mercury (II) residues with the in situ rapid nondestructive imaging in simulated fresh agricultural products. Thus, this PSM strategy could provide good guidance for environmental protection methodologies in the future.

## 1. Introduction

Rapid industrialization has released significant amounts of pollutants, including heavy metal ions, into the environment. Among the most toxic and hazardous contaminants, mercury is extensively used in batteries, barometers, float valves, thermometers, and other devices [1]. However, when introduced into natural water environments through microbial processes, ionic mercury readily converts into neurotoxic methylmercury. Even trace levels of mercury bioaccumulation can lead to organ and health issues, such as digestive, kidney, and nervous system disorders [2]. Therefore, the detection of mercury ions in aqueous solutions is of paramount importance. Numerous techniques have been employed to detect mercury, including atomic absorption spectrometry (AAS), cold vapor atomic fluorescence spectroscopy (AFS), mass spectrometry (MS), and liquid chromatography (LC) [3,4]. However, these methods encounter challenges related to intricate sample preparation and costly equipment, which restrict their extensive in-field application. Consequently, achieving prompt response and the highly selective and sensitive detection of Hg^2+^ in water remains a formidable challenge.

Metal–organic frameworks (MOFs) [5,6,7] materials have garnered significant attention in the last two decades due to their high surface area, exceptional stability, adjustable porosity, customizable chemical functionality, and potential applications, particularly in gas storage and separation [8,9], heterogeneous catalysis [10,11], chemical sensing [12,13,14,15], proton conduction [16], and drug delivery [17]. Specifically, as fluorescent sensors, MOFs offer the advantage of precise structural design and control by selecting different metal ions and organic ligands to meet the needs of various analytes. Simultaneously, they can selectively adsorb molecules with specific sizes or properties through the sieving action of their pores, thereby concentrating the analytes within MOFs. This can enhance the sensitivity of detecting target molecules. Some fluorescent sensors based on MOFs for detecting Hg^2+^ have been documented [18,19,20]. For example, Wen et al. developed two amino-decorated MOFs for the selective and quantitative detection of Hg^2+^, attributed to the chelating interaction between Hg^2+^ and the pendant amino motif of the ligand [21]. Our group reported the first example of novel dual-emission MOF-implicated ratiometric sensor for mercury (II) in pure water with an LOD of about 2 nM, attributed to Hg^2+^-induced structural collapse [22]. More recently, Wang et al. designed and synthesized several FRET-based MOFs with different donor/acceptor ratios. The MOF with an optimized donor/acceptor ratio of 7.0 was employed as an efficient fluorescence turn-on sensor for Hg (II) ions, demonstrating good sensitivity and selectivity [23].

However, certain challenges, such as the compatibility of the functional groups, limited linker solubility, and inadequate chemical, or thermal stability in some cases, can hinder direct synthesis. Moreover, there are several factors that restrict their widespread use, including their inability to function in pure water, susceptibility to interference from other metal ions, and the inability to achieve precise metal ion detection specificity. Cohen and Burrows, among others, have established that the post-synthetic modification (PSM) of MOFs can yield new active sites as an effective and flexible strategy [24,25]. This approach enhances selectivity and augments detection sensitivity, offering a promising solution to the aforementioned problems. As an example, Lang and his colleagues utilized PSM in a two-dimensional metal–organic framework through photodimerization. The resulting PSM product displayed the capability to detect Al^3+^ ions through a luminescent quenching mechanism, offering significantly enhanced selectivity and sensitivity [26].

In this study, we focus on the exceptional water stability of UiO-series MOFs, attributed to the robust interaction between zirconium (IV) (Zr^4+^) clusters and carboxylic ligands [27]. To this end, we synthesized a conventional UiO-66-type MOF (UiO-66-NH_2_) utilizing a microwave-assisted method, which yields primary amine groups suitable for subsequent post-synthetic functionalization. Through the post-synthetic modification of UiO-66-NH_2_ with 2-(Methylthio)benzaldehyde, a novel sulfur-containing MOF (UiO-66-NSMe) was created. Notably, when tested in the HEPES buffer, UiO-66-NSMe exhibited a significantly improved ability to selectively detect Hg^2+^ compared to the original compound. Furthermore, we delve into the mechanistic understanding behind this enhanced selectivity. The in situ imaging of UiO-66-NSMe for mercury (II) ion detection has also been developed.

## 2. Materials and Methods

### 2.1. Materials

All chemicals used in this study were of reagent-grade quality and were obtained from commercial sources without the need for additional purification. Specifically, 2-aminoterephthalic acid (99%) and acetic acid (99.5%) were purchased from the Shanghai Aladdin Biochemical Technology Co., Ltd. (Shanghai, China). N,N-Dimethylformamide (DMF, 99.5%), methanol (99.5%), Zirconium (IV) chloride (ZrCl_4_, 98%), 2-(Methylthio)benzaldehyde (97%), and HEPES (99%) were purchased from Beijing Innochem Science & Technology Co., Ltd. (Beijing, China). Mercury (II) nitrate hydrate (98%) and the other metal salts were purchased from Shanghai Fourth Chemical Reagent Company (Shanghai, China). Stock solution (2 × 10^−2^ M) of the aqueous nitrate salts of K^+^, Na^+^, Li^+^, Cd^2+^, Zn^2+^, Mn^2+^, Fe^2+^, Cu^2+^, Ba^2+^, Sr^2+^, Ca^2+^, Mg^2+^, Ni^2+^, Co^2+^, Pb^2+^, Ag^+^, Fe^3+^, Cr^3+^, Al^3+^, and Hg^2+^ were prepared for further experiments. Deionized water generated from the purification chain was used for all experiments. Lettuce was purchased at the Zhaishan Farmers’ Market (No. 8 Chengnan Avenue, Quanshan District, Xuzhou, China).

### 2.2. Characterization Methods

The X-ray powder diffraction (XRD) patterns of UiO-66-NH_2_, UiO-66-NSMe, and UiO-66-NSMe after Hg^2+^ detection were recorded on a Bruker D8 Advance X-ray powder diffractometer using Cu–K*α* (λ = 1.5405 Ǻ) radiation. FT-IR spectra were recorded as KBr pellets on Bruker Optics TENSOR 27 FT-IR spectrophotometer (Bruker, Billerica, MA, USA). X-ray photoelectron spectroscopy (XPS) experiments were performed using a PHI QUANTUM2000 surface analysis instrument. The morphologies of the prepared samples were recorded by a Field Emission Scanning Electron Microscopy (SEM) by Hitachi (SU8010) (Tokyo, Japan). Samples were treated via Pt sputtering for 90 s before observation. Nitrogen sorption isotherms were recorded on an Autosorb-IQ2 instrument at 77 K. ^1^H NMR spectra were recorded using Me_4_Si as an internal standard on a Bruker-400 spectrometer. The Zr^4+^ contents before and after Hg^2+^ detection were measured by Inductively Coupled Plasma Spectrometer (Perkin Elmer, Waltham, MA, USA). The solution fluorescence spectra were measured on JASCO FP6500. Both excitation and emission slit widths were 5 nm, and fluorescence measurements were carried out in a 1 cm quartz cuvette while stirring the suspension of UiO-66-NH_2_ or UiO-66-NSMe.

### 2.3. Synthesis of UiO-66-NH_2_

Following the methodology outlined in the references [28], the synthesis of UiO-66-NH_2_ was achieved through a microwave-assisted procedure. Initially, a combination of 2-aminoterephthalic acid (0.75 mmol, 0.135 g) and ZrCl_4_ (0.75 mmol, 0.175 g) was introduced into a solution composed of DMF (20 mL) and acetic acid (2.4 mL). This mixture was then subjected to microwave reactor and heating at 120 °C for a duration of 1 h. After cooling to room temperature, the solution underwent centrifugation, resulting in a precipitate that underwent a thorough washing process using DMF and MeOH, which was repeated three times, to effectively remove any unreacted ligand/metal. The resulting gray-colored powder was collected and subsequently air-dried.

### 2.4. Synthesis of UiO-66-NSMe

A quantity of UiO-66-NH_2_ weighing 100 mg was dispersed within a solution of 2-(Methylthio)benzaldehyde (1.2 mmol, 0.178 g) in MeOH (50 mL), along with two drops of acetic acid. The resulting mixture was subjected to stirring at 75 °C for a duration of 24 h. Following the completion of the reaction, the resultant product, UiO-66-NSMe, was obtained in a colorless form. The obtained products were collected through a filtration process, subsequently washed with MeOH, and finally air-dried.

### 2.5. Digestion and ^1^H NMR on UiO-66-NSMe

Approximately 10 mg of UiO-66-NSMe materials was desiccated under vacuum at 100 °C for 8 h. Subsequently, it were subjected to digestion by d^6^-DMSO (500 μL) and 48% HF (30 μL) via sonication for 1 h until a clear solution was achieved. The resulting solution was then subjected to ^1^H NMR analysis.

### 2.6. Detection of Hg^2+^

In the typical procedure, 1 mg of finely ground UiO-66-NSMe sample was carefully dispersed in 3 mL of HEPES buffer with a pH of 7.4. Following a 5 min sonication period to ensure thorough mixing, the initial fluorescence spectra of UiO-66-NSMe were recorded. Subsequently, the fluorescence spectra of UiO-66-NSMe were measured upon the addition of Hg^2+^ ions, using an excitation wavelength of 360 nm. To assess the selectivity of UiO-66-NSMe for Hg^2+^, similar experiments were conducted with various other metal ions, each undergoing a comparable sensing procedure in the solution.

In the analysis of Hg^2+^ in spiked samples, we employed lake water, tap water, and rainwater devoid of Hg^2+^ as representative sample matrices. Spiked lake water, tap water, and rainwater were subjected to direct detection immediately following spiking. To simulate the direct detection of Hg^2+^ residue on the surfaces of garden produce, we sprayed lettuce samples with solutions containing varying concentrations of Hg^2+^. After a 2 min interval, a UiO-66-NSMe water suspension was applied to the smeared surfaces, followed by another 2 min wait period. Subsequently, we observed the samples under UV light. To prepare the fluorescent test paper, a standard light-yellow wood-colored filter paper was cut into a square measuring 2 cm in both length and width. Subsequently, the filter paper was immersed in a UiO-66-NSMe solution and allowed to air-dry naturally.

### 2.7. Typical Procedure for Cyanosilylation of Aldehydes

Into a 3 mL CH_2_Cl_2_ mixture containing 1.2 mmol of Trimethylsilyl cyanide ((CH_3_)_3_SiCN, TMSCN) and 0.5 mmol of aromatic aldehyde, UiO-66-NSMe or UiO-66-NSMe⊃Hg^2+^ (5 mg) was introduced. The resulting mixture was stirred at room temperature for a duration of 3 h. The progression of the reaction was monitored via thin-layer chromatography (TLC), and conversions were quantified through ^1^H-NMR analysis.

## 3. Results

### 3.1. Characterization of PSM Product UiO-66-NSMe

**Figure 1:**
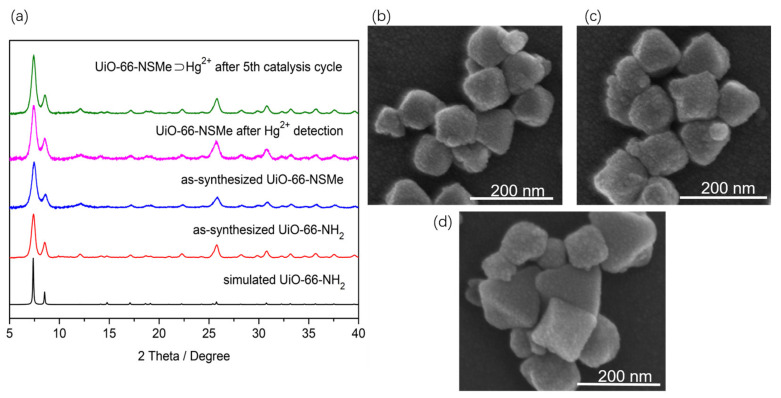
(**a**) Powder X-ray diffraction (XRD) profiles for the simulated (black) and as-synthesized UiO-66-NH_2_ (red), as-synthesized UiO-66-NSMe (blue), UiO-66-NSMe after Hg^2+^ detection (magenta), and UiO-66-NSMe⊃Hg^2+^ after the fifth catalysis cycle (olive). (**b**) SEM imaging of UiO-66-NH_2_. (**c**) SEM imaging of UiO-66-NSMe. (**d**) SEM imaging of UiO-66-NSMe after Hg^2+^ detection.

A reaction involving ZrCl_4_ and 2-aminoterephthalic acid in DMF utilizing microwave synthesis yielded UiO-66-NH_2_. The resulting material exhibited powder X-ray diffraction (PXRD) patterns that were notably congruent with those previously reported for the UiO-66 topology (Figure 1a). Additionally, scanning electron microscopy (SEM) observations displayed a consistently uniform octahedral morphology (Figure 1b), aligning with the findings in the existing literature [29,30], all confirming the successful fabrication of UiO-66-NH_2_. Subsequently, sulfur-containing functional groups were covalently incorporated into the framework by exploiting Schiff base reactions between amino groups and aldehyde groups within the framework structure.

By conducting a reflux reaction involving UiO-66-NH_2_ and 2-(Methylthio)benzaldehyde with glacial acetic acid as the catalyst, UiO-66-NSMe was obtained. The synthesized material underwent filtration, washing, and drying steps. The PXRD patterns demonstrated that the structural integrity of UiO-66-NMSe remained unchanged during the process of post-synthetic modification (Figure 1a). Simultaneously, the morphology of the samples was examined using SEM. As depicted in Figure 1c, the images of UiO-66-NSMe exhibited a uniformly dispersed octahedral structure. These observations are in line with the PXRD analysis, indicating that the PSM process applied to UiO-66-NH_2_ did not exert a discernible influence on its microstructure.

**Figure 2:**
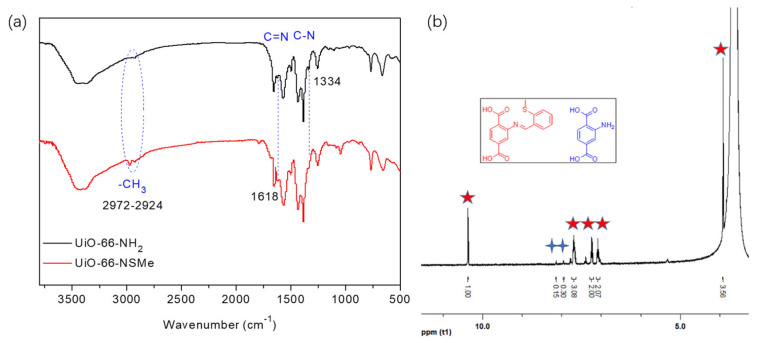
(**a**) FT-IR spectra of UiO-66-NH_2_ and UiO-66-NSMe. (**b**) ^1^H NMR spectra of UiO-66-NSMe digested in DMSO-d_6_/HF.

The successful synthesis of UiO-66-NSMe was subjected to a comprehensive analysis. A comparison of the FT-IR spectra of UiO-66-NSMe with UiO-66-NH_2_, depicted in Figure 2a, revealed new peaks at approximately 2972–2924 cm^−1^. These peaks correspond to the characteristic C-H stretching vibrations of the methyl group in UiO-66-NSMe. Importantly, a new peak appeared at 1618 cm^−1^, which is indicative of the characteristic C=N stretching vibrations specific to UiO-66-NSMe. Simultaneously, the peak at 1334 cm^−1^, which was attributed to the C-N stretching vibration of UiO-66-NH_2_, nearly disappeared. The alterations in the FT-IR spectra collectively demonstrated that the structure of UiO-66-NSMe is characterized by the absence of free amino groups, replaced by the creation of new C=N groups via the Schiff base reaction. In addition, methyl groups associated with -SMe are unambiguously identified within the structure of UiO-66-NSMe.

Another solid evidence supporting the formation of Schiff base reaction products is derived from ^1^H NMR studies. In DMSO-d_6_/HF, UiO-66-NSMe displayed a resonance signal at 10.37 ppm, corresponding to the –CH=N- proton, together with a methyl proton signal at 3.92 ppm. The integration area between these signals is consistent with the anticipated product, affirming the success of the PSM process (Figure 2b). A modification rate of up to 87% was determined by calculating the ratio of aromatic region at PSM products (Figure 2b, red star) to the proton signal of 2-aminoterephthalic acid (Figure 2b, blue cross) in ^1^H NMR after the digestion of UiO-66-NSMe. Furthermore, the XPS full spectra of UiO-66-NSMe (Figure 4) unveiled a new peak at 163.07 eV in the S 2p region, providing further confirmation of successful PSM and the resultant formation of UiO-66-NSMe. At the same time, the elemental composition obtained by XPS for UiO-66-NSMe indicated that PSM extent is calculated as 84%, which is quite close to the ^1^H NMR result (Table S1).

**Figure 3:**
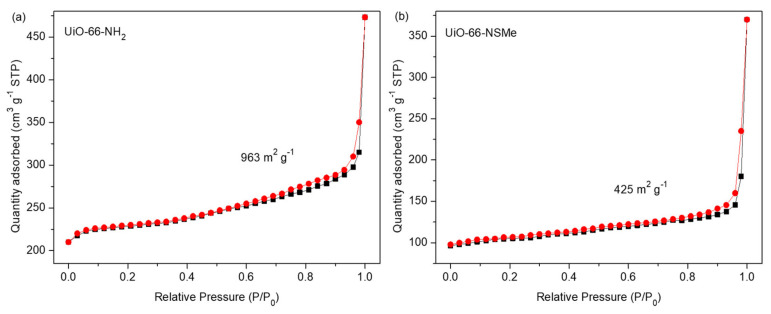
N_2_ sorption isotherms for compounds UiO-66-NH_2_ (**a**) and UiO-66-NSMe (**b**) at 77 K. The adsorption and desorption branches are shown with black square and red circle, respectively.

N_2_ sorption isotherms were subsequently measured at 77 K on the as-synthesized UiO-66-NH_2_ and UiO-66-NSMe samples. It was observed that the surface area had decreased from 963 m^2^ g^−1^ to 425 m^2^ g^−1^, further confirming the successful formation of PSM products (Figure 3). Nonetheless, UiO-66-NSMe still maintains its pores, ensuring the ingress and egress of guest molecules or ions. All these data collectively highlight the successful covalent post-synthetic modification of UiO-66-NH_2_, in which a Schiff base reaction was effectively established between the amino group within the MOF and the aldehyde group, all while maintaining the structural integrity.

### 3.2. Luminescent Detection of Metal Ions in HEPES Buffer Based on UiO-66-NSMe

**Figure 4:**
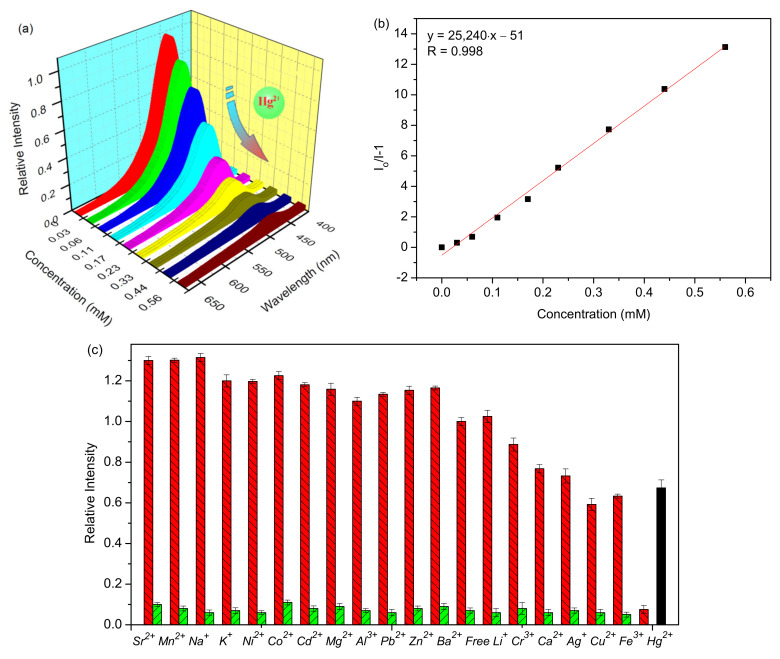
(**a**) Luminescence spectra of UiO-66-NSMe upon incremental addition of Hg^2+^ up to 0.56 mM in HEPES buffer, with excitation at 360 nm. (**b**) Stern–Volmer plot of UiO-66-NSMe quenched by Hg^2+^. (**c**) The red bars stand for the luminescence intensities of UiO-66-NSMe when the selected metal ions (1.12 mM) are present. The green ones stand for the change of the emission when continuing to add 0.56 mM Hg^2+^ (The black bar represents the fluorescence intensity of UiO-66-NH_2_ upon adding 0.56 mM of Hg^2+^).

Considering the presence of sulfur atoms within the UiO-66-NSMe framework, we have delved into its metal-ion-sensing capabilities, particularly its exploitation of the thiophilic nature of mercury ions [31,32,33]. Under excitation at 360 nm, the UiO-66-NSMe suspension in HEPES buffer exhibited a pronounced fluorescence emission peak at approximately 450 nm (Figure 4a). Luminescence titrations involving Hg^2+^ were then conducted with the UiO-66-NSMe suspension to assess its capacity for detecting mercury (II) ions. Notably, rapid and substantial fluorescence quenching was observed upon the addition of Hg^2+^ solution to the UiO-66-NSMe suspension, reaching concentrations up to 0.56 mM (Figure 4a). This quenching effect can be quantified using the Stern–Volmer equation, I_0_/I = 1 + *K*_sv_[M], where I denotes the luminescence intensity after Hg^2+^ addition, I_0_ is the initial luminescence intensity of UiO-66-NSMe, [M] represents the molar concentration of the introduced Hg^2+^, and *K*_sv_ signifies the Stern–Volmer constant. Through calculation based on the experimental data in Figure 3a, a *K*_sv_ value of 2.5 × 10^4^ M^−1^ was determined (Figure 4b). Following the 3δ IUPAC criteria, the limit of detection for Hg^2+^ is approximately 20 nM. Therefore, UiO-66-NSMe proves to be a proficient material for detecting mercury (II) ions, with its heightened sensitivity for Hg^2+^ attributed to the affinity-driven interaction between Hg^2+^ and the sulfur atoms present.

To assess the selectivity of UiO-66-NSMe towards Hg^2+^, its luminescence responses were examined upon the introduction of various other metal ions, including Cr^3+^, Al^3+^, K^+^, Co^2+^, Sr^2+^, Cd^2+^, Na^+^, Mg^2+^, Ba^2+^, Pb^2+^, Ni^2+^, Cu^2+^, Zn^2+^, Ag^+^, Ca^2+^, Fe^3+^, Li^+^, and Mn^2+^ (Figure 4c and Figure S1). The fluorescence spectra of UiO-66-NSMe exhibited minimal changes in the presence of K^+^, Co^2+^, Mg^2+^, Ba^2+^, Pb^2+^, Ni^2+^, Al^3+^, Li^+^, Cd^2+^, and Zn^2+^. Slight fluorescence enhancement was observed with Sr^2+^, Mn^2+^, and Na^+^. In UiO-66-NSMe, the fluorescence quenching induced by Cr^3+^, Ca^2+^, Ag^2+^, Cu^2+^, and Fe^3+^ was only 40% of their highest quenching efficiency. In sharp contrast, the addition of Hg^2+^ ions (0.56 mM) led to a fluorescence quenching exceeding 93%. Comparatively, the introduction of Hg^2+^ ions into the original UiO-66-NH_2_ suspension resulted in only a 33% fluorescence quench. Meanwhile, the anti-interference capability of UiO-66-NSMe for Hg^2+^ detection was further explored by competing experiments (Figure 4c, green bars). The experimental result reveals that the coexistence of interferers had no obvious interference on the Hg^2+^ detection. This distinction highlights the remarkable selectivity of UiO-66-NSMe for Hg^2+^. The framework modification through PSM, leading to the introduction of sulfur (S) moieties, emerges as an effective approach to enhance the detection of mercury (II) ions.

To further validate the detection mechanism of Hg^2+^ on UiO-66-NSMe, the PXRD patterns of the UiO-66-NSMe samples following Hg^2+^ detection matched well with the original pattern (Figure 1a). This alignment demonstrates that the primary framework structure remained unchanged. Moreover, inductively coupled plasma (ICP) measurements revealed minimal Zr^4+^ content present in the filtrate (Table S2), effectively excluding the possibilities of compound dissolution and the exchange of Hg^2+^ and Zr^4+^ within the lattice, which could lead to the drastic luminescence quenching observed. These findings are additionally supported by the sustained SEM morphology even after detecting Hg^2+^ ions (Figure 1d). Collectively, these results reinforce the notion that the significant luminescence quenching in response to Hg^2+^ is not attributed to structural changes or dissolution but rather is due to specific interaction between Hg^2+^ and UiO-66-NSMe.

**Figure 5:**
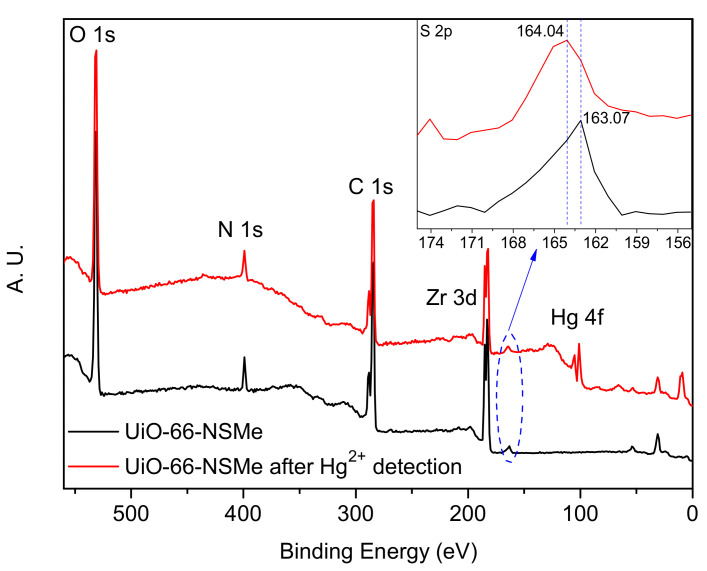
XPS spectra of UiO-66-NSMe before and after Hg^2+^ detection.

Furthermore, the X-ray photoelectron spectroscopy (XPS) spectra of UiO-66-NSMe were measured both before and after Hg^2+^ detection. After Hg^2+^ detection, a new peak corresponding to Hg 4f at a binding energy of approximately 100.91 eV emerged in the full spectrum of UiO-66-NSMe. This unequivocally confirms the adsorption of Hg^2+^ within the channels of UiO-66-NSMe (Figure 5). Intriguingly, a shift from 163.07 eV to 164.04 eV in the binding energy of the S 2p peak was observed before and after Hg^2+^ detection. Such a shift implies an augmented electron density around the sulfur (S) atoms, likely stemming from the coordination interaction between Hg^2+^ and S atoms within UiO-66-NSMe. Thus, combined with the structure of UiO-66-NSMe, it is possible that Hg^2+^ ions are chelating coordinated by the S and N sites in UiO-66-NSMe.

As the UiO-66-NSMe detection of Hg^2+^ relies on the coordination between mercury and sulfur sites, the material can be recycled after detecting Hg^2+^. This newly coordinated Hg^2+^ material, donated as UiO-66-NSMe⊃Hg^2+^, possesses additional metal centers compared to the original material, thereby enhancing its Lewis acidic properties. In its role as a catalyst, UiO-66-NSMe⊃Hg^2+^ demonstrates improved catalytic activity for Lewis acid-catalyzed cyanosilane reactions. To illustrate this enhancement, we conducted a model reaction using benzaldehyde and (CH_3_)_3_SiCN at room temperature in CH_2_Cl_2_ for 3 h. With UiO-66-NSMe⊃Hg^2+^ (5 mg) as catalysts, the expected product, 2-phenyl-2-((trimethylsilyl)oxy)acetonitrile, was obtained in >99% yield. In contrast, when UiO-66-NSMe was used as the catalyst, it yielded a 58% 2-phenyl-2-((trimethylsilyl)oxy)acetonitrile with no observed byproducts. This substantial difference can be attributed to the enhanced Lewis acid properties conferred by UiO-66-NSMe⊃Hg^2+^.

Importantly, UiO-66-NSMe⊃Hg^2+^ could be readily recovered from the catalytic system through centrifugation and subsequently reused in fresh conditions. Remarkably, this catalyst exhibited no significant decrease in efficiency; the yield for 2-phenyl-2-((trimethylsilyl)oxy)acetonitrile was 97% for the second run, 98% for the third run, 96% for the fourth run, and 97% for the fifth run. The stability of UiO-66-NSMe⊃Hg^2+^ was confirmed by XRD after the fifth catalysis cycle (Figure 1a). This exceptional catalytic cycling performance further underscores the robust interaction between UiO-66-NSMe and mercury (II) ions, reinforcing the previous findings of coordination between mercury (II) ions and sulfur sites.

With these impressive catalytic results in hand, we extended our investigation to explore the generality and scope of the current protocol across a variety of aromatic aldehydes in conjunction with (CH_3_)_3_SiCN. As presented in Table 1 (Figures S2–S7), when utilizing UiO-66-NSMe⊃Hg^2+^ as the catalyst, the yields for 4-methoxybenzaldehyde, 4-nitrobenzaldehyde, and 4-chlorobenzaldehyde reached 96%, 95%, and 93%, respectively. These yields were almost twice as high as when UiO-66-NSMe was used as the catalyst. However, as the size of the substrates increased, catalytic efficiency exhibited a substantial decrease. For instance, 1-naphthaldehyde yielded only 51%, while the larger 3,5-di-tert-butylbenzaldehyde resulted in a mere 15% yield when UiO-66-NSMe⊃Hg^2+^ served as the catalyst. These outcomes underscore that Hg^2+^-modified UiO-66-NSMe performs well not only in the cyanosilylation of aromatic aldehydes but also displays promising size-selective behavior. This presents a bright prospect for the design of more active catalyst materials and offers an effective means for the reutilization of test materials.

### 3.3. In situ Imaging Detection of Hg^2+^ in Real Water, Vegetables, and Test Paper

To evaluate the reliability for practical Hg^2+^ detection by using the prepared material in real water samples, we employed a “turn-off” fluorescent sensor based on UiO-66-NSMe. Given that Hg^2+^ ions were not naturally present in rainwater, lake water (Yunlong Lake, Yunlong district, Xuzhou, China), or tap water (No. 1 Beijing South Road, Tongshan district, Xuzhou, China), we conducted recovery experiments using spiked samples. The added amounts of Hg^2+^ were set at 10 μM, 20 μM, and 30 μM, respectively. The fluorescence-based method for detecting Hg^2+^, relying on UiO-66-NSMe in real water samples, as indicated in Table 2 (Figures S8 and S9), yielded recovery rates ranging from 96.1% to 99.5%. These outstanding detection results demonstrate that our designed UiO-66-NSMe possesses the capability of reliable detection for Hg^2+^ in real-world samples.

**Figure 6:**
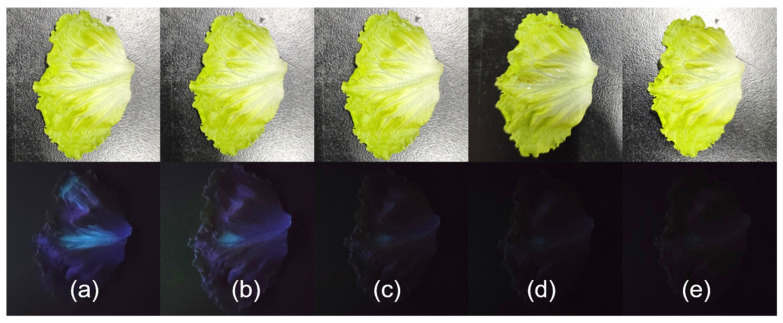
Noticeable differences in fluorescence became evident when UiO-66-NSMe suspension (0.33 g/L) was introduced to the surface of the lettuce under ultraviolet light, and the change in fluorescence was observed within 30 s. Lettuce was subjected to spraying with solutions of varying concentrations: 0 (**a**), 0.04 (**b**), 0.18 (**c**), 0.35 (**d**), and 0.56 (**e**) mM Hg^2+^, simulating mercury (II) residue.

Given UiO-66-NSMe’s remarkable ability to rapidly and efficiently detect Hg^2+^, we conducted further investigations to assess its potential for on-site detection. UiO-66-NSMe was employed to simulate the detection of mercury (II) residue on the surface of garden produce through direct imaging technology. Because vegetables are sensitive agricultural products that require a lot of water to irrigate, exogenous mercury (II) ions can enter them. Since Hg^2+^ ions were not naturally present on the lettuce we obtained, we simulated mercury (II) contamination by applying varying concentrations of Hg^2+^ solution to their surfaces. Subsequently, luminescence was observed under UV light at 365 nm following the addition of the UiO-66-NSMe suspension. As illustrated in Figure 5, a significant reduction in UiO-66-NSMe luminescence on the surface of lettuce was observed, progressively diminishing until complete quenching as the Hg^2+^ concentration increased from 0.04 mM to 0.56 mM (Figure 6). Collectively, these results strongly indicate that UiO-66-NSMe holds great promise for the rapid and on-site detection of mercury (II) residues in real samples through direct imaging techniques.

**Figure 7:**
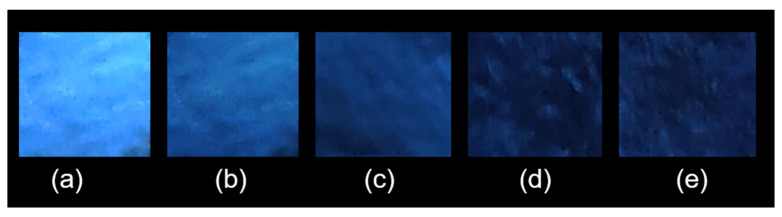
Photographs depicting the fluorescence quenching of UiO-66-NSMe on test strips for the visual detection of small amounts of Hg^2+^ under 365 nm UV light: (**a**) test strip; Hg^2+^ of different concentrations (**b**) 0.04 mM, (**c**) 0.18 mM, (**d**) 0.35 mM, (**e**) 0.56 mM.

To facilitate simple and portable detection, we developed a fluorescence test paper for the rapid assessment of Hg^2+^ in aqueous solutions. This test paper was created by immersing a light-yellow wood-colored Whatman filter paper measuring 2.0 × 2.0 cm^2^ into a dispersion of ground UiO-66-NSMe in water and subsequently allowing it to dry at room temperature. For the detection of minuscule quantities of Hg^2+^ ions, 1 μL of aqueous solutions containing varying concentrations of Hg^2+^ ions was placed onto the UiO-66-NSMe test papers. As depicted in Figure 7, when exposed to UV light at 365 nm, the test papers exhibited varying colors of different intensities that were discernible to the naked eye, corresponding to the concentration levels of Hg^2+^ ions. The minimum detectable amount of Hg^2+^ ions was calculated to be at the remarkably low level of 13.7 ng. All these practice experimental findings underscore the advantages of UiO-66-NSMe as an efficient, sensitive, and selective sensor for Hg^2+^ ions.

## 4. Conclusions

In conclusion, we have successfully synthesized a novel framework with the -SCH_3_ functional group, designated as UiO-66-NSMe, through post-synthetic modification (PSM). The effective conversion was verified by FT-IR, XPS, and ^1^H NMR analyses, yielding a PSM rate of up to 87%. Luminescence-based studies of metal ion detection demonstrated that UiO-66-NSMe displays exceptional selectivity and sensitivity towards Hg^2+^ in HEPES buffer. Additionally, the comprehensive detection mechanism was attributed to the presence of a coordination interaction between Hg^2+^ and S atoms within UiO-66-NSMe. The MOF sensor was also utilized for the detection of Hg^2+^ in tap water, rainwater, and river water spiked with the contaminant. Additionally, it was employed to detect mercury (II) residues with in situ rapid nondestructive imaging in simulated fresh agricultural products. Moreover, a straightforward and portable test paper enabled the visual detection of Hg^2+^ ions at the nanogram level by the naked eye. We believe that this study holds promise for advancing environmental detection methodology in the future.

## Figures and Tables

**Table 1:** Results of the cyanosilylation reaction catalyzed by UiO-66-NSMe and UiO-66-NSMe⊃Hg^2+^.

Entry	Substrates	Yields (%)	
		UiO-66-NSMe⊃Hg^2+^	UiO-66-NSMe
1	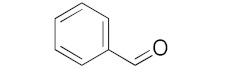	>99	58
2	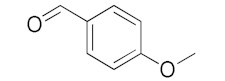	96	55
3	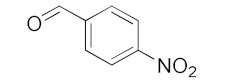	95	52
4	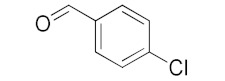	93	54
5	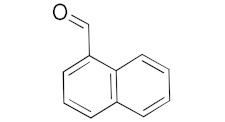	51	33
6	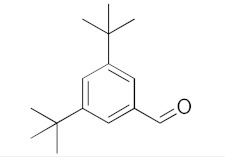	15	6

Reaction conditions: aromatic aldehydes (0.50 mmol), catalyst (5 mg), (CH_3_)_3_SiCN (1.2 mmol), CH_2_Cl_2_ (3 mL) are added to the cuvette. The cuvette is stirred at room temperature for 3 h.

**Table 2:** Spike and recovery test of Hg^2+^ by using UiO-66-NSMe in real water samples.

Samples	Spiked (μM)	Found (μM)	Recovery (%)
Rainwater	10	9.65	96.5
	20	19.52	97.6
	30	29.34	97.8
Lake water	10	9.95	99.5
	20	19.78	98.9
	30	28.83	96.1
Tap water	10	9.84	98.4
	20	19.64	98.2
	30	29.12	97.1

## Data Availability

All data are contained within the article.

## Supplementary Materials

The following supporting information can be downloaded at: https://www.mdpi.com/article/10.3390/nano13202784/s1, Table S1: XPS elemental compositions (atomic %); Figure S1: The fluorescence spectra of UiO-66-NSMe in HEPES buffer upon the addition of 0.56 mM of various metal ions; Figures S2 to S7: ^1^H NMR characterization of catalytic products by as-synthesized UiO-66-NSMe⊃Hg^2+^; Table S2: The ICP result on the filtrate of UiO-66-NSMe after Hg^2+^ detection; Figure S8: The relative fluorescence intensity of UiO-66-NSMe with varying Hg^2+^ concentrations; Figure S9: Spike test of Hg^2+^ by using UiO-66-NSMe in real water samples.
