# Supplementary material for: Post-Synthetic Modification of an Amino-Functionalized Metal–Organic Framework for Highly In Situ Luminescent Detection of Mercury (II)
Source: Nanomaterials (Basel). 2023 Oct 17;13(20):2784. doi: 10.3390/nano13202784 (PMC10610009; doi:10.3390/nano13202784)
Supplement: Supplementary file 1 [file nanomaterials-13-02784-s001.zip › nanomaterials-2634041-supplementary.pdf]

Supporting Information for

Post-Synthetic Modification of an Amino-Functionalized Met-al–Organic Framework for Highly In Situ Luminescent Detection of Mercury (II)

Chen Ji, Li Pei, Junyi Qin, Pengyan Wu *, Nuo Su, Ting Zhang, Yexin Zhang and Jian Wang *

Jiangsu Key Laboratory of Green Synthetic Chemistry for Functional Materials, School of Chemistry and Materials Science, Jiangsu Normal University, Xuzhou 221116, China.

Table S1. XPS elemental compositions (atomic %).

Entry	Atomic %						Chemical Formula	
	Zr	O	C	N	S	Hg	Exp.	Calcd.
UiO-66-NH_2	6.51	34.56	52.41	6.52			$ZrC_{8.05}O_{5.31}N$	$ZrO_{0.66}(OH)_{0.66}(C_8O_4H_3NH_2)$
UiO-66-NSMe	4.35	23.39	64.23	4.37	3.66		$ZrC_{14.77}O_{5.38}NS_{0.84}$	$[ZrO_{0.66}(OH)_{0.66}(C_8O_4H_3NH_2)]_{0.16}\cdot[ZrO_{0.66}(OH)_{0.66}(C_{16}O_4H_{11}NS)]_{0.84}$
UiO-66-NSMe⊃Hg^{2+}	4.21	22.65	61.8	4.25	3.50	3.59	$ZrC_{14.68}O_{5.38}NS_{0.83}Hg_{0.85}$	

For UiO-66-NSMe, the chemical formula is $(ZrC_{8.05}O_{5.31}N)_{0.16}\cdot(ZrC_{16.05}O_{5.39}NS)_{0.84}$, thus, the PSM extent was calculated as 84%, which is consistent with the result of ^1H NMR (87%).

Figure S1. The fluorescence spectra of UiO-66-NSMe in HEPES buffer upon the addition of 0.56 mM of various metal ions.

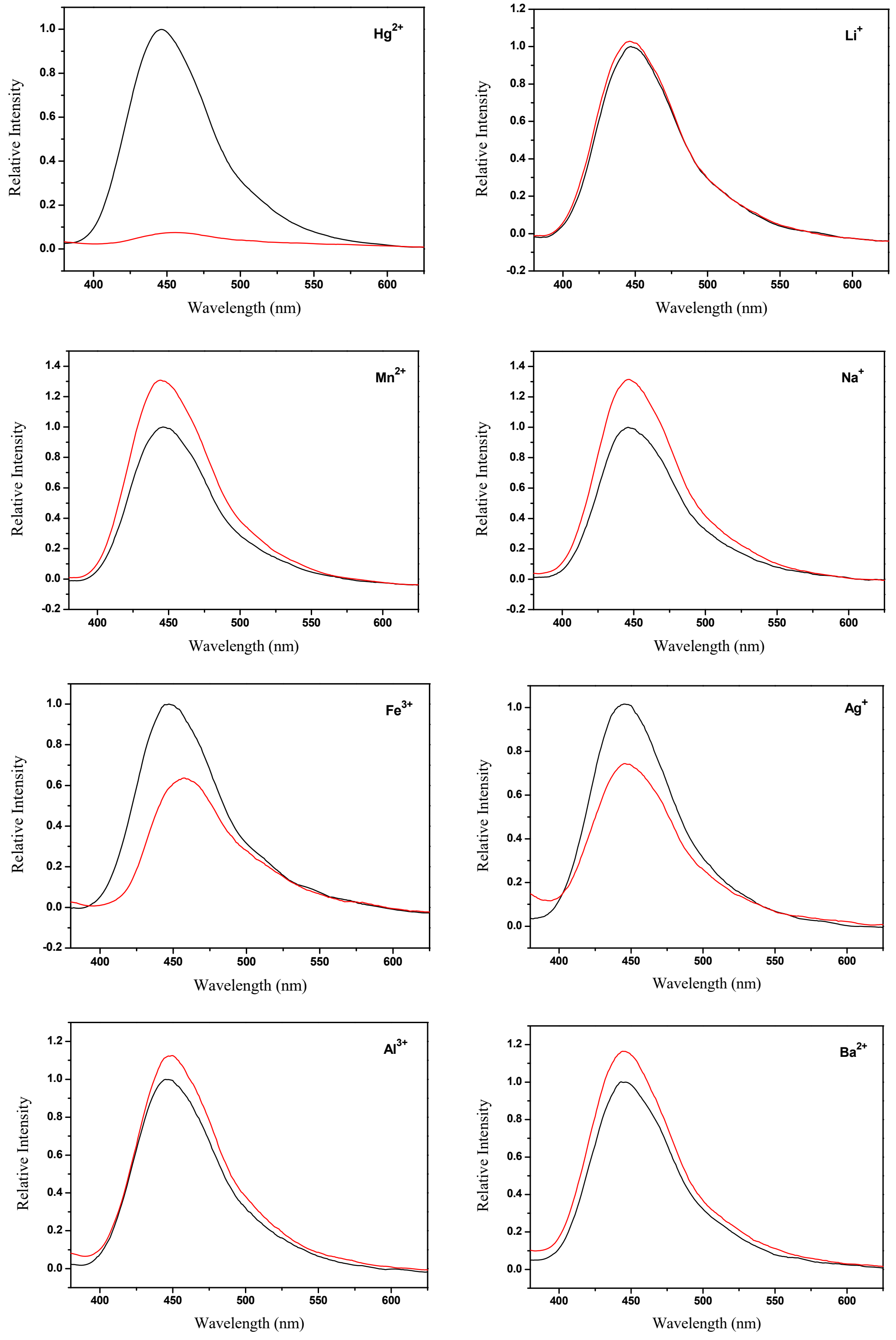

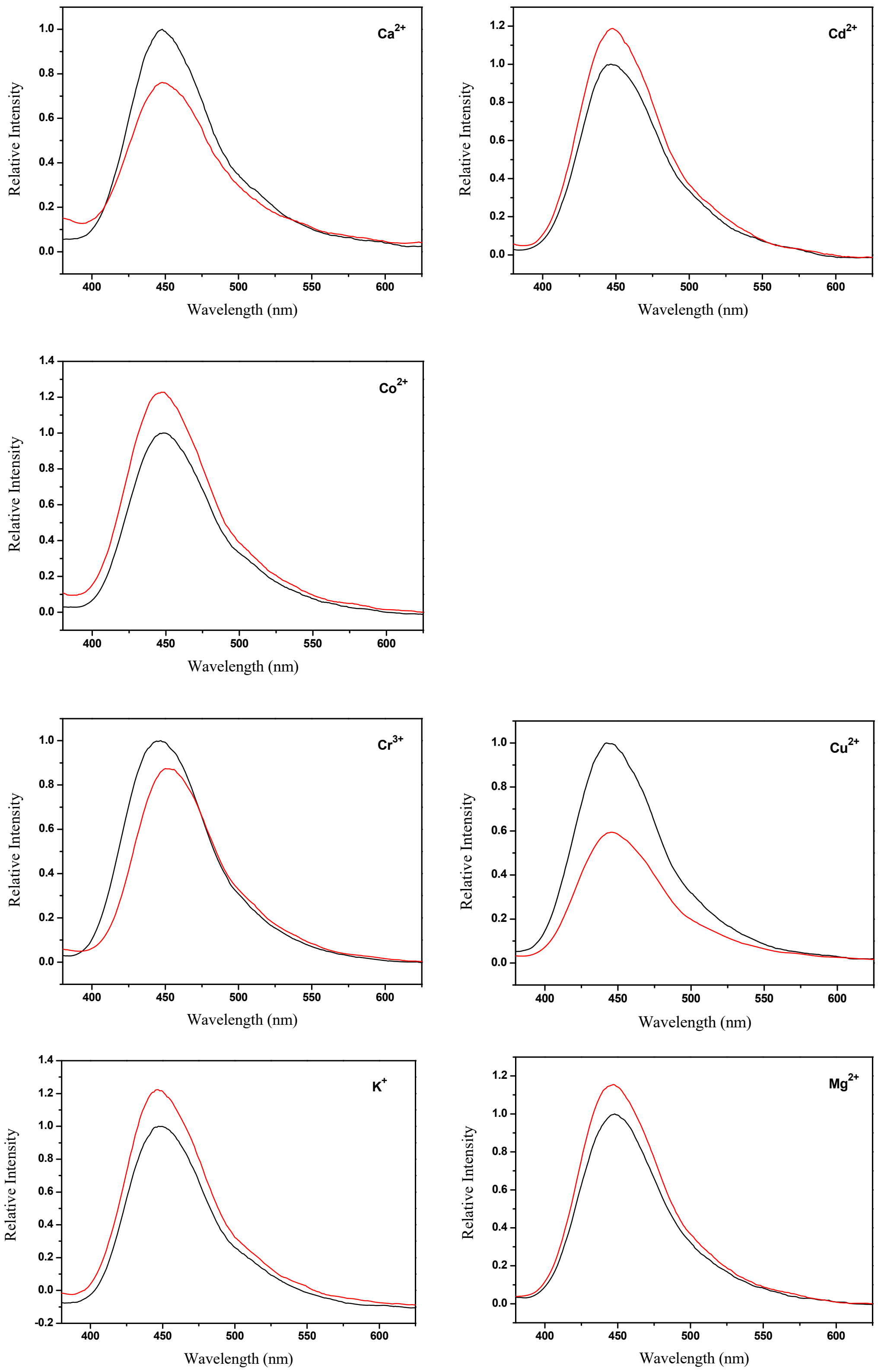

Ca2+
Cd2+
Co2+
Cr3+
Cu2+
K+
Mg2+
Relative Intensity
Wavelength (nm)

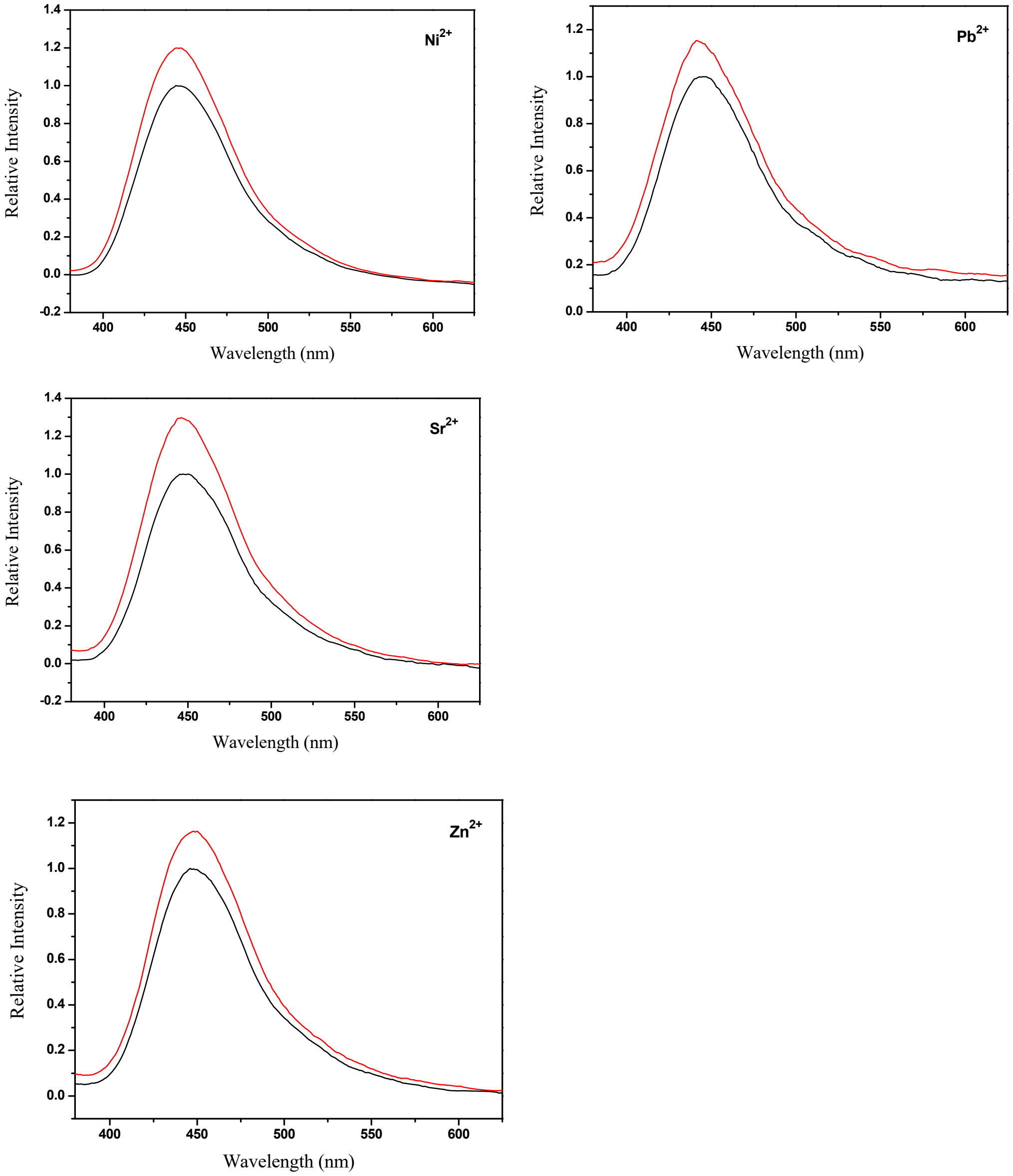

Ni2+
Pb2+
Sr2+
Zn2+
Relative Intensity
Wavelength (nm)

^{1}H NMR Characterization of catalytic products by as-synthesized UiO-66-NSMe⊃Hg^{2+}.

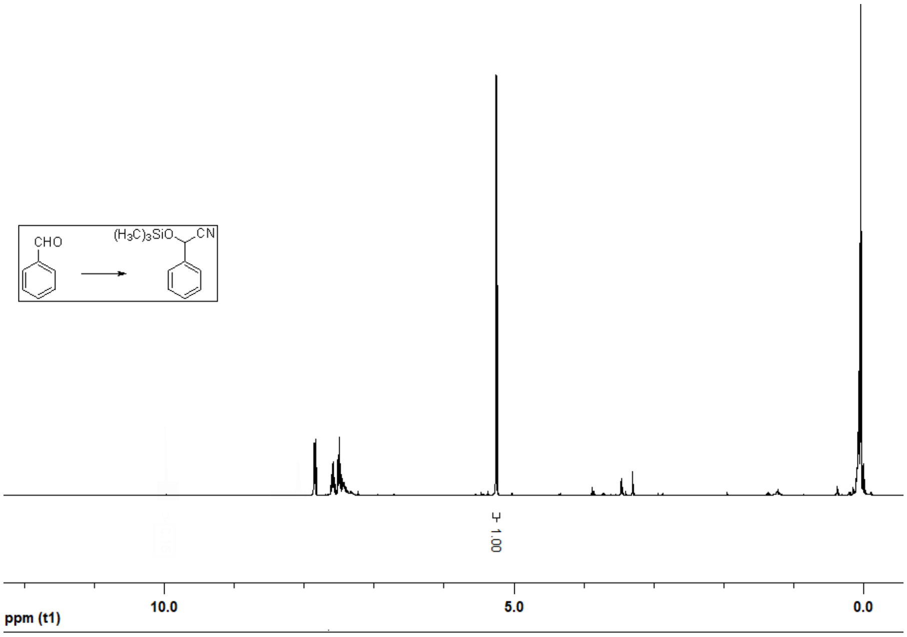

Figure S2. ^{1}H NMR (400 MHz, $CDCl_3$) of 2-phenyl-2-(trimethylsilyloxy)-acetonitrile.

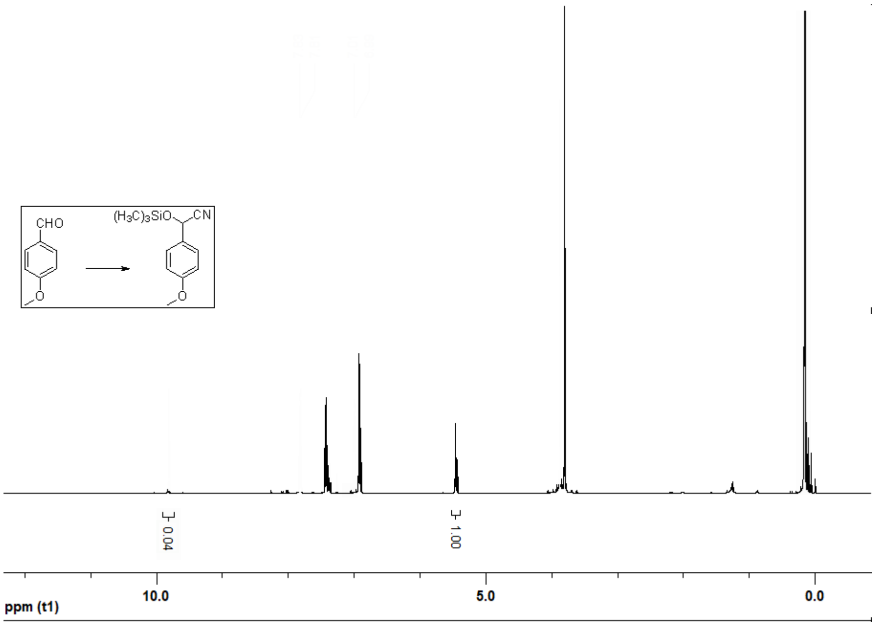

Figure S3. ^{1}H NMR (400 MHz, $CDCl_3$) of 2-(4-methoxyphenyl)-2-(trimethylsilyloxy)-acetonitrile.

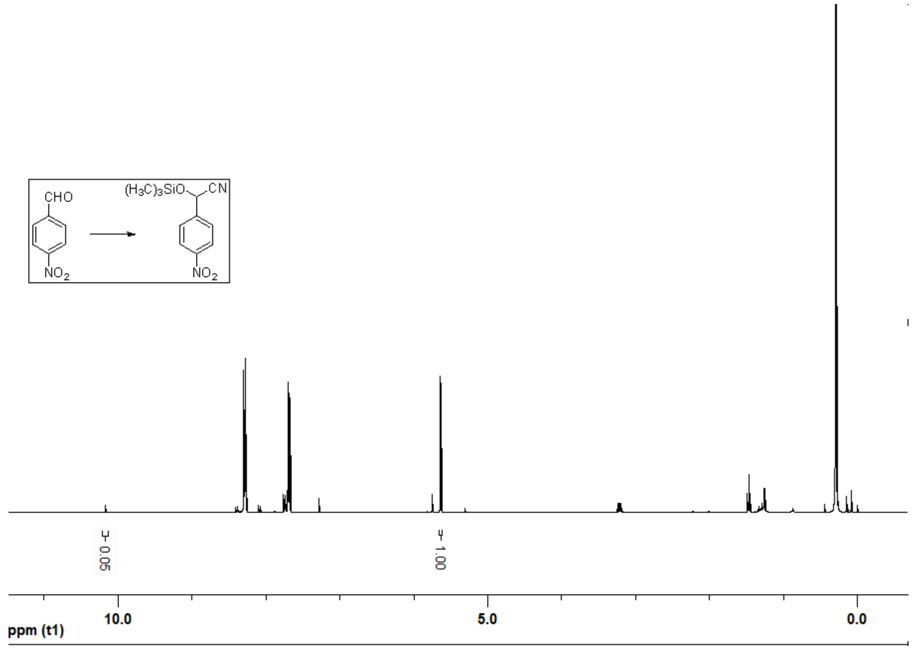

Figure S4. ^{1}H NMR (400 MHz, $CDCl_3$) of 2-(4-nitrophenyl)-2-((trimethylsilyl)oxy)-acetonitrile.

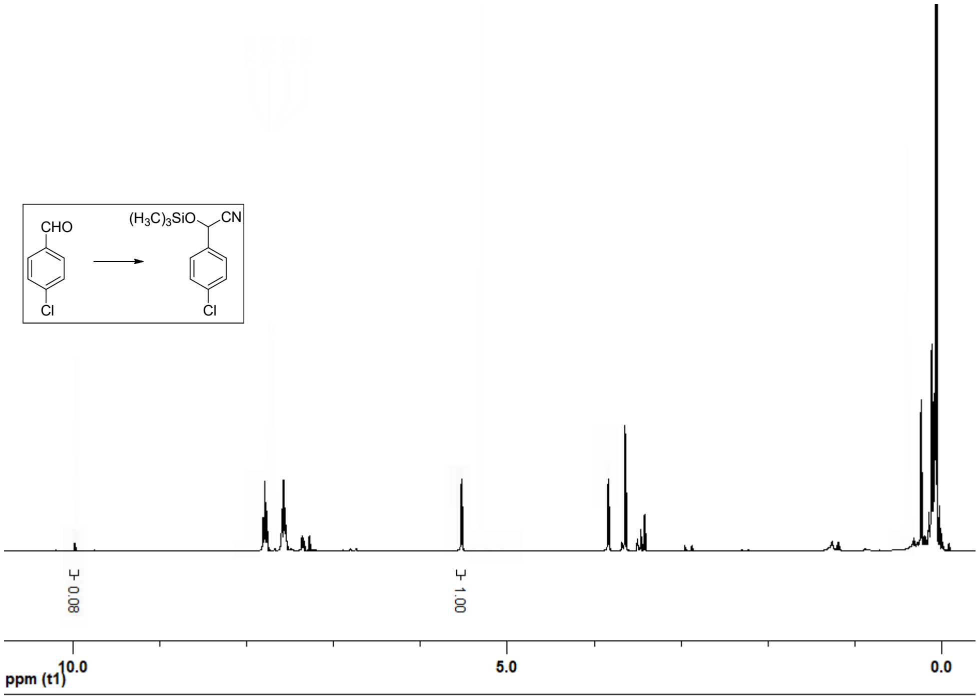

Figure S5. ^{1}H NMR (400 MHz, $CDCl_3$) of 2-(4-chlorophenyl)-2-((trimethylsilyl)oxy)acetonitrile.

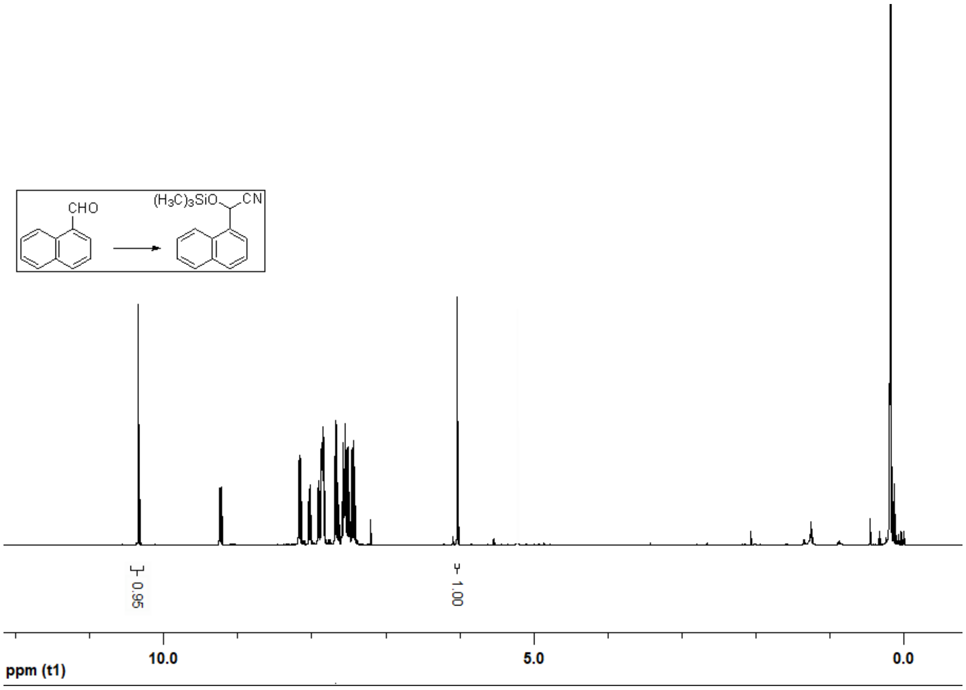

Figure S6. ^{1}H NMR (400 MHz, $CDCl_3$) of 2-(naphthalene-1-yl)-2-(trimethylsilyloxy)-acetonitrile.

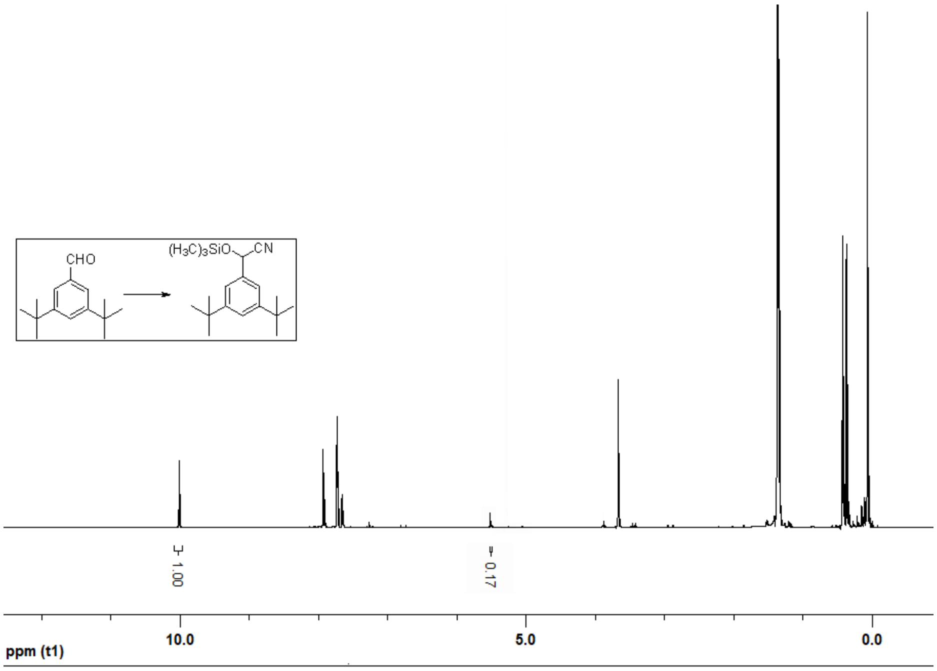

Figure S7. ^{1}H NMR (400 MHz, $CDCl_3$) of 2-(3,5-di-tert-butylphenyl)-2-((trimethylsilyl)oxy)acetonitrile.

Table S2. The ICP result on the filtrate of UiO-66-NSMe after Hg^{2+} detection.

	$[Zr^{4+}]$ (μg/mL)
Filtrate	0.005 ± 0.0003

Figure S8. The relative fluorescence intensity of UiO-66-NSMe with varying Hg^{2+} concentrations.

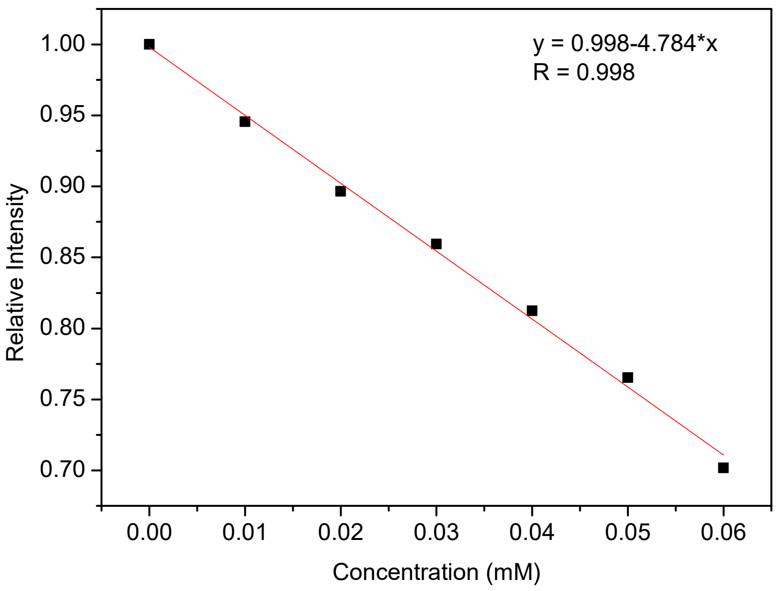

Figure S9. Spike test of Hg^{2+} by using UiO-66-NSMe in real water samples.

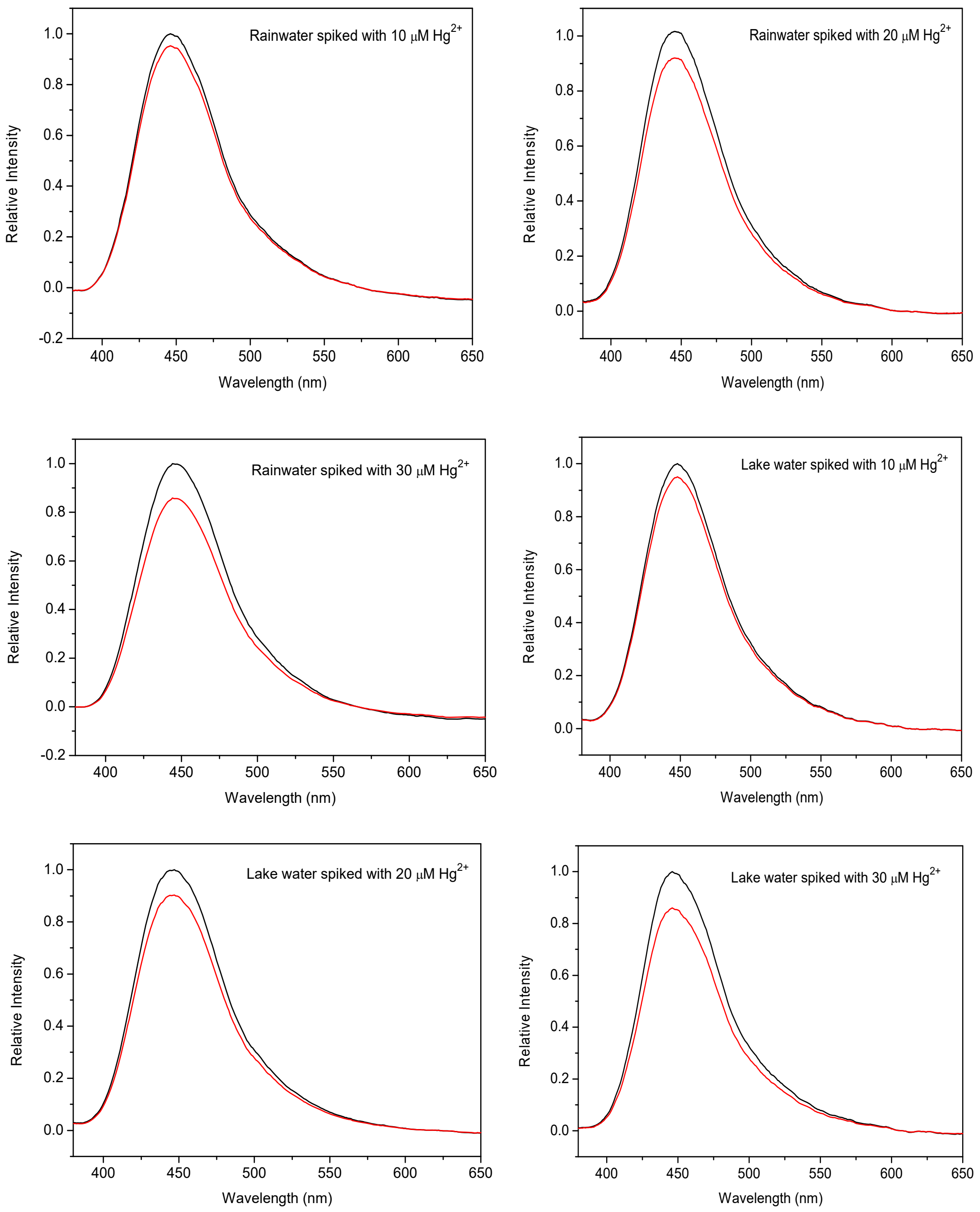

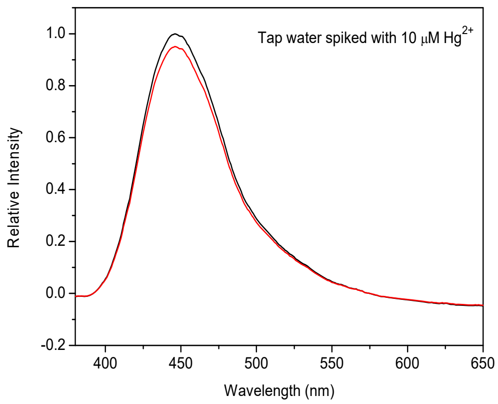
Tap water spiked with 10 μM Hg2+
Relative Intensity
Wavelength (nm)
1.0
0.8
0.6
0.4
0.2
0.0
-0.2
400
450
500
550
600
650

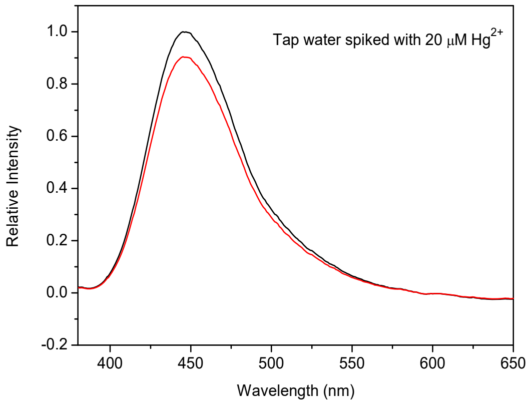
Tap water spiked with 20 μM Hg2+
Relative Intensity
Wavelength (nm)
1.0
0.8
0.6
0.4
0.2
0.0
-0.2
400
450
500
550
600
650

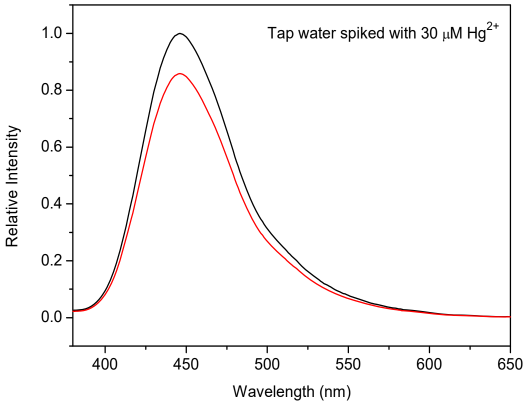
Tap water spiked with 30 μM Hg2+
Relative Intensity
Wavelength (nm)
1.0
0.8
0.6
0.4
0.2
0.0
400
450
500
550
600
650
